# Land Cover Change in the Central Region of the Lower Yangtze River Based on Landsat Imagery and the Google Earth Engine: A Case Study in Nanjing, China

**DOI:** 10.3390/s20072091

**Published:** 2020-04-08

**Authors:** Dong-Dong Zhang, Lei Zhang

**Affiliations:** 1 Shanghai Key Laboratory of Multidimensional Information Processing, East China Normal University, Shanghai 201100, China; 52161213021@stu.ecnu.edu.cn; 2 MOE International Joint Lab of Trustworthy Software, East China Normal University, Shanghai 200062, China

**Keywords:** land-use/cover change, Google Earth Engine, spatiotemporal analysis, driving mechanism, Nanjing

## Abstract

Urbanization in China is progressing rapidly and continuously, especially in the newly developed metropolitan areas. The Google Earth Engine (GEE) is a powerful tool that can be used to efficiently investigate these changes using a large repository of available optical imagery. This work examined land-cover changes in the central region of the lower Yangtze River and exemplifies the application of GEE using the random forest classification algorithm on Landsat dense stacks spanning the 30 years from 1987 to 2017. Based on the obtained time-series land-cover classification results, the spatiotemporal land-use/cover changes were analyzed, as well as the main factors driving the changes in different land-cover categories. The results show that: (1) The obtained land datasets were reliable and highly accurate, with an overall accuracy ranging from 88% to 92%. (2) Over the past 30 years, built-up areas have continued to expand, increasing from 537.9 km^2^ to 1500.5 km^2^, and the total area occupied by built-up regions has expanded by 178.9% to occupy an additional 962.7 km^2^. The surface water area first decreased, then increased, and generally showed an increasing trend, expanding by 17.9%, with an area increase of approximately 131 km^2^. Barren areas accounted for 6.6% of the total area in the period 2015–2017, which was an increase of 94.8% relative to the period 1987–1989. The expansion of the built-up area was accompanied by an overall 25.6% (1305.7 km^2^) reduction in vegetation. (3) The complexity of the key factors driving the changes in the regional surface water extent was made apparent, mainly including the changes in runoff of the Yangtze River and the construction of various water conservancy projects. The effects of increasing the urban population and expanding industrial development were the main factors driving the expansion of urban built-up areas and the significant reduction in vegetation. The advantages and limitations arising from land-cover mapping by using the Google Earth Engine are also discussed.

## 1. Introduction

The urbanization of the contemporary world is continuously increasing, especially in developing countries, where urbanization is progressing at an unprecedented rate and scale. Along with the development of industrialization and urbanization, population and wealth are further concentrated in large cities, and new urban spatial organization forms have gradually emerged, such as a megalopolis [1], metropolitan area (MA), mega-urban region, and urban agglomeration [2]. Among them, MA refers to the regional economy phenomenon that appears in the urban agglomeration with a big city as the core and the surrounding cities participating in the division of labor, cooperation, and integration. Since the reform and opening up of China in 1978, the process of urbanization has accelerated [3], and increasingly more MAs have emerged.

With the constant expansion of MAs in China, the built-up area has increased dramatically, which inevitably has had a major impact on other land-use/land-cover (LULC) categories for limited land resources. China’s per capita cultivated land was much less than the average level of the world in 2012 [4], and the shortage of urban water resources was also increasingly prominent [5]. The contradiction between the development demands of urbanization and the carrying capacity of land resources is increasingly important. Under the impetus of economic development and population growth, the irrational development and utilization of land resources are gradually overstretching the carrying capacity of the urban ecological environment, creating a potential threat to the sustainable development of human society. This requires the establishment of effective government regulations on land use to realize sustainable economic development. Therefore, quantifying, analyzing, and monitoring the long-term spatial and temporal dynamics of urban LULC is essential for better understanding urban land surface processes, as well as urban land administration and planning.

The accurate and efficient mapping of urban land use requires high-resolution imagery, improved classification and change detection algorithms, and powerful platforms [6,7]. Satellite-based remote sensing is the primary approach for detecting long-term land-cover change and it has helped us acquire valuable earth observation information by providing large numbers of remotely sensed images [8]. While high spatial resolution (<5 m) satellite images (e.g., SPOT5, IKONOS-2, and QuickBird) provide abundant and detailed ground features, the available data only dates back to the late 2000s. The Landsat series of satellites has provided the longest record (from 1972 to the present) of continuous and freely available imagery for the entire globe (e.g., Goward et al. [9]), allowing for long-term urban change monitoring, which is an invaluable resource for monitoring global change and a primary source of medium spatial resolution (5–100 m) earth observations used in decision-making [10,11,12,13,14,15]. In general, significant changes in urban land cover are found at annual to decadal timescales [16]. Furthermore, the ideal situation for monitoring land-cover change is to obtain the remote sensing data within an extended range (15–30 days) on different timescales in the same region. However, the availability of satellite imagery is unpredictable due to issues including high cloud cover and data gaps, which result in large time differences in image acquisition (e.g., seasonal differences or even more), which may lead to large errors in land-cover classification and assessing regional scale land-cover change. To address this issue, the dense time stacks method proposed by Schneider essentially used a “brute force” approach to place all relatively cloud-free Landsat scenes in a single composite dataset, where gap areas and clouds were not masked or corrected in any way during processing [17]. By ignoring the use of annual data for change detection, dense stacks utilize all available Landsat scenes intersecting the study area, including images with relatively high cloud cover that is subsequently masked out, leaving the cloudless part to participate in the stacking process. In addition, maps of known features and calculated vegetation indices suitable for the development of a more nuanced classification scheme can be incorporated [18].

Over the past decade, many approaches for land classification algorithms have been explored, including pixel-based (image classification, regression, etc.), sub-pixel based, object-oriented algorithms, spectral mixture analysis (SMA), random forest (RF), and artificial neural networks (ANNs) [19,20,21]. Due to the different model parameters and environments given by different research areas, it is difficult to determine which classification algorithm is the most common and/or most widely applicable. Among these algorithms, SMA and RF are two widely used methods for detecting urban land-cover change. SMA was developed based on the vegetation-impervious surface-soil (V-I-S) model [22], where three endmembers—vegetation, impervious surface, and soil—are chosen to model heterogeneous urban compositions [23], except for urban water bodies. The RF model is a classifier that uses multiple trees to train and predict samples and has been widely used in many applications. Recently, a free, web-based computing platform called the Google Earth Engine (GEE) [24] has provided access to global time-series satellite imagery and other ancillary data (e.g., vector data, digital elevation models, and weather and climate data) and algorithms for processing large amounts of data with relative ease. In addition, parallel analysis in the GEE enables many processors to participate in any given computation, thus greatly speeding up the process compared to desk computing [25]. It has already been used in various applications, including mapping vegetation cover [26,27,28], settlement and population [29], and the detection of the boundaries of urban areas [30]. Previous studies have been limited by computing power and storage, and they have used limited multispectral bands as inputs in RF models; however, with the computational power of the GEE, multidimensional feature data can now be introduced into RF models, such as the calculated indexes (e.g., the normalized difference vegetable index (NDVI) [31], the normalized difference water index (NDWI) [32], and the normalized difference built-up index (NDBI)), digital elevation model (DEM), and many other factors related to land cover. In addition, the traditional method used for training-sample selection was the visual interpretation of Google Earth or high-resolution remote-sensing data. When it comes to the large regional or even national scales, the entire process requires extensive labor and time, which needs the efficient and rapid construction of a training sample dataset.

Supported by the GEE, we performed a multi-date composite change detection technique by utilizing dense stacks of Landsat imagery and used the expanded RF algorithm to monitor urban LULC change based on the constructed sample datasets with the available auxiliary data. The objectives of this study were (1) to propose a methodology for accurately and quickly monitoring long-term urban land-cover changes using the GEE; (2) to analyze the dynamics of LULC changes in the core city of the Nanjing MA in the central region of the lower Yangtze River over the past 30 years, especially changes in urban surface hydrology, vegetation, and built-up area; and (3) to explore the influence of the meteorological environment and socioeconomic factors on urban land-cover changes through multiple linear regression analysis.

## 2. Materials and Methods

### 2.1. Study Area

**Figure 1:**
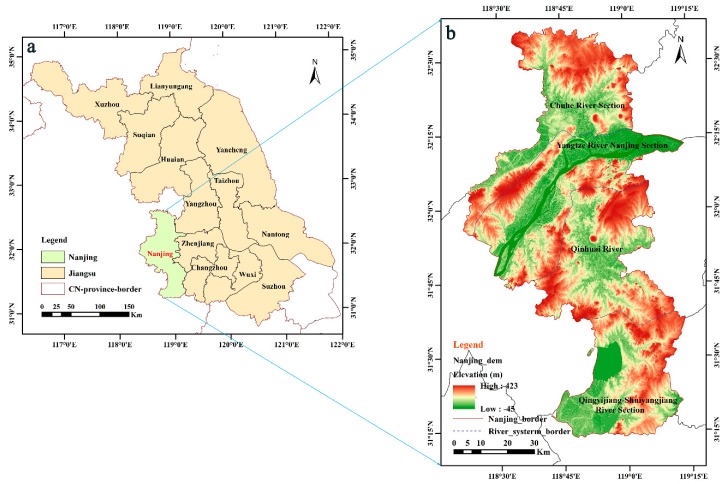
An overview of the study area: (**a**) the location of Nanjing in China; (**b**) digital elevation model (DEM) and divided water system of the study area. Shuttle Radar Topography Mission (SRTM) data source: http://srtm.csi.cgiar.org.

The study area, located in the central region of the lower Yangtze River, is representative of the lower Yangtze River region and southwest capital of Jiangsu Province, as shown in Figure 1. Nanjing, as the core city of the Nanjing MA, is an important gateway city for the Yangtze River Delta for driving the development of the central and western regions. The longitude ranges from 118.35° E to 119.233° E and the latitude ranges from 31.236° N to 32.611° N. The Yangtze River runs through the city, with a total length of nearly 200 km. There are 120 large and small rivers in the territory, belonging to four rivers (Yangtze River, Qingyijiang River-Shuiyangjiang River, Chuhe River, and Qinhuai River) and two lakes (Gucheng Lake, Shijiu Lake), which was divided into four water systems: the Yangtze River Nanjing Section, the Chuhe River, the Qinhuai River, and the Qingyijiang River-Shuiyangjiang River. Nanjing has a subtropical humid climate with an annual average temperature of 15.4 °C, and the annual average precipitation is 1106 mm. The rainy season lasts from late June to early July every year. Nanjing city has 11 districts with a total area of 6587 km^2^. The resident population was 8.335 million and the urbanization rate reached 82.3% in 2017. Nanjing has become the only megacity in the Yangtze River delta and east China.

**Figure 2:**
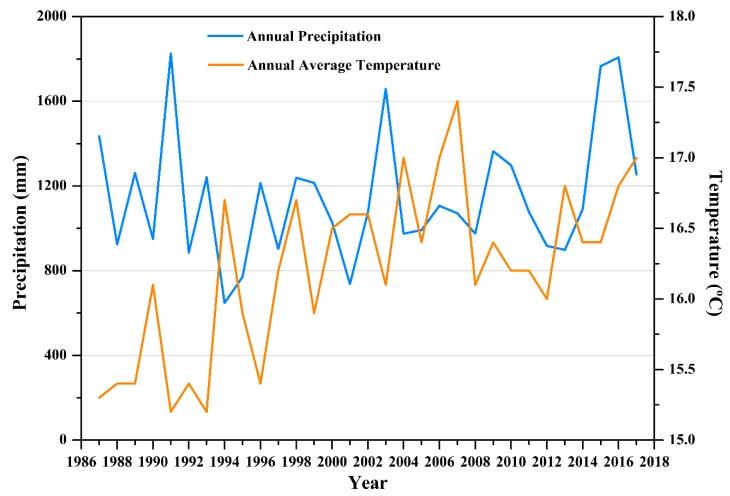
Average annual precipitation and average temperature changes in the study area.

The average annual precipitation and average temperature of the study area from 1987 to 2017 are shown in Figure 2. For the study area, the meteorological environment is complicated because of its vulnerability to East Asian summer monsoons, Pacifica subtropical highs, typhoons, and rainy seasons, and the precipitation data showed obvious seasonal and inter-annual variations. Raw meteorological data were taken from the National Meteorological Information Center (http://data.cma.cn).

### 2.2. Data Acquisition and Processing

The Landsat series of satellites have provided continuous imagery for the past few decades. These images are contained in Google Earth Engine’s public data archive and can be used to detect and estimate long-term dynamic land-cover change. The Landsat images were directly selected and processed on the Google Earth Engine platform. The entirety of Landsat 5, 7, and 8 image collections was analyzed using the Tier 1 top of atmosphere (TOA) reflectance product for the study area with the written application of multi-scene image display, spanning from 1987 to 2017. The details of the reflectance product and the constants for all the Landsat sensors (Thematic Mapper (TM), Enhanced Thematic Mapper (ETM+), Operational Land Imager (OLI)) were described and tabulated in Chander et al. [15]. The auxiliary data, which included the Shuttle Radar Topography Mission (SRTM) digital elevation dataset [33], Defense Meteorological Satellite Program-Operational Linescan System (DMSP-OLS) nighttime imagery [34], and other open-source vector data from Open Street Map (OSM) (www.openstreetmap.org) were available in the Google Earth Engine and used as supporting information for selecting the training samples to improve the classification accuracy. In addition, the climate and socioeconomic data derived from the TerraClimate [35] dataset, the Yangtze River Yearbook and Sediment Bulletin, and Nanjing statistical yearbook were analyzed to explore the main factors driving changes in regional land-cover types.

**Figure 3:**
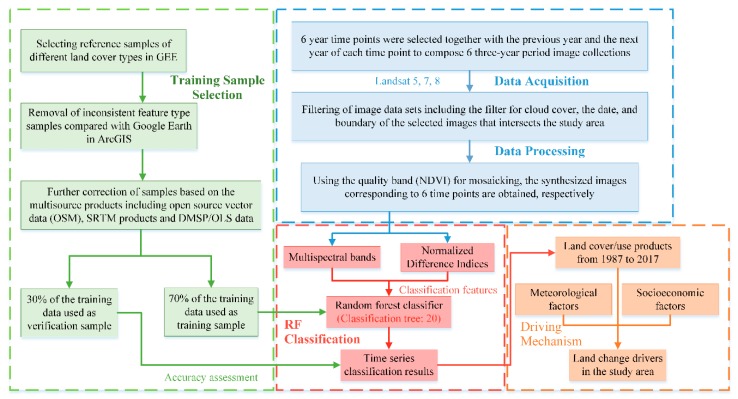
Flowchart of land-cover mapping and the mechanism analysis. Land-cover mapping included the data acquisition and processing, training-sample selection, and random forest (RF) classification.

The image processing steps for mapping the land-cover change of the study area are summarized in Figure 3. In this study, we developed a procedure that involved compositing the available images intersecting the study area in three-year periods to produce a cloud-free composite image, selected based on the cloud coverage which was and the climate factor within the study area. The first step was the development of a cloud-masking function by assigning a cloud score to each pixel in the image collections. A cloud threshold of 25 was selected based on the visual interpretation of Landsat imagery [36] with the built-in algorithm “ee.Algorithms.Landsat.simpleCloudScore,” which computed a cloud-likelihood score to compare multiple views of the same point for a relative cloud likelihood [37], and pixels with a cloud-score higher than 25 were masked out. To remove the impact of the rainy season and snow cover on the classification result, the date frame was set from 1 March to 15 June and 16 July to 31 November each year. Based on the availability of filtered images, six image collections were created with each image collection to account for data gaps. The corresponding time nodes were 1988, 1993, 1998, 2004, 2009, and 2015, respectively. Each time node, together with the previous year and the following year, constituted a three-year period; detailed information is given in Table 1. Furthermore, all data from Landsat 7 after 31 May 2003 were abnormal because of the sudden failure of the Landsat 7 ETM+ Scan Lines Corrector (SLC) which needed to be corrected. Here, the written GapFill function in the script was used for the correction.

The filtered image collections were acquired after the cloud mask and limited date range were applied. In addition, the NDVI, NDBI, NDWI, and modified normalized difference water index (MNDWI) [38] were calculated using the internal function “normalizedDifference” and added as bands to the images within the image collections.

Taking the uneven inter-annual and seasonal precipitation into consideration, the next step was to use the quality bands for mosaicking, i.e., the pixel with the highest value in the quality band would be used in the resulting mosaic. In this work, the NDVI was used as the quality band for the generation of the primary mosaic with the algorithm “qualityMosaic,” which composited all the images in the collections using the NDVI band as a per-pixel ordering function. That is, “qualityMosaic()” sorted the pixels by the NDVI band and returned the other bands’ values corresponding to the maximum in the NDVI band such that the created mosaic was a composite of the “greenest” pixels and called the greenest image, which depicted the greatest extent of vegetation with minimal surface water extent [39,40]. Using this strategy, bare land could be easily distinguished from vegetation, and the contours and shapes of the surface water extent obtained were comparable with the permanent surface water of the Joint Research Centre (JRC) Yearly Water Classification History, which can be regarded as permanent surface water [8,41]. To reduce storage and to focus on the study area, the created mosaics were clipped afterward to only include the area of Nanjing. This process resulted in six composites of Landsat images with surface spectral information and the created quality bands that would be used for further classification and long-term dynamic LULC detection.

### 2.3. Methodology

#### 2.3.1. Technical Process

The technical process consisted of four steps:

- Based on the GEE platform, six quality composites were acquired via image date filtering, cloud masking, and mosaicking, spanning from 1987 to 2017, as described in Section 2.2.
- Normalized difference indexes were utilized in the training-sample selection. With the support of multisource land-cover products, training and verification sample points were carefully deployed according to the “complete consistency” and “temporal stability” principles. The land-cover attributes were then extracted for the samples.
- The RF model was applied for extracting land-cover types based on Landsat satellite images and auxiliary data with the selected training samples. The mapping of the land cover in Nanjing was performed by applying the training rules.
- The mechanisms of land-cover change with infrastructure construction and human activities in this area were explored with multiple stepwise regression modeling, combined with climate change and social development factors.

The overall technical process is shown in Figure 3.

#### 2.3.2. Training-Sample Selection

Normalized difference indexes were used to select the training samples more accurately and effectively. The exact vegetation information was preferentially extracted and masked out for its obvious difference in spectral characteristics with other land-cover types. After checking the effects of many repeated experiments, the threshold values of NDVI greater than or equal to 0.5 and MNDWI less than −0.3 were set to extract and mask out the vegetation information; this procedure could be easily carried out with the code “updateMask(ndvi.it(0.5) and (mndwi.gte(−0.3)))” (“ndvi” and “mndwi” are the new bands added to the image by the former step). A preliminary non-vegetation map was acquired by applying the vegetation mask, then training samples for other land-cover types could be more easily and effectively selected within a reduced range. For the periods of 2003–2005, 2008–2010, and 2015–2017, the training sample points/polygons representing the ground object types were interpreted by referring to the high-resolution imagery available on Google Earth, of which the time range corresponding to that of the classified composite image. For the periods before 2000, sample sites were selected based on the context relation and the historical topographic maps available for the study area. A total of 1099, 1123, 1507, 1124, 1071, and 1347 training sample points were selected for each composite image, respectively. Open-source vector data acquired from OSM were used as validation data dating from 2014. Additionally, the SRTM products and DMSP-OLS data were used as reference data to improve the classification accuracy. Among these sample points, 70% were gathered as training samples during classification. The remaining points were used to verify the accuracy of the classification results.

#### 2.3.3. Landsat Classification and Accuracy Assessment

The obtained composites were then classified within the GEE using the RF classification method with the following four land-cover categories: (1) water, including river, lake, reservoir, pond, mudflat, and wetland; (2) vegetation, including cropland, woodland, and grassland; (3) built-up area, including urban and rural areas, industrial and mining land, and residential land; and (4) barren, mainly including bare land, saline-alkali land, and bare rock gravel land.

The RF classification method uses the bootstrap resampling technique (otherwise known as bagging) to build an ensemble of classification and regression-tree (CART) classifiers within training polygons representing the spectral signatures of various land-cover classes [42]. The algorithm not only randomly selects sub-samples from the input variables, but also randomly selects the best feature through a voting process to establish the splits in the nodes of trees (which reduces the likelihood of overfitting). Among all the current algorithms, the RF algorithm produces excellent accuracies and can run effectively on large data sets [18].

In the RF model, the number of decision trees and features at each node for splitting are vital to the classification results [43]. In this study, RF classifiers with different trees were applied and the classification tree was set to 30 based on the overall accuracy and kappa coefficient, as shown in Supplementary Figure S1. Furthermore, the data input into the RF model included the original spectral bands (bands B1–B5 and B7 for Landsat 5/7, and B2–B6 and B8 for Landsat 8), combined with the four calculated normalized indices. Moreover, the classification results of different land-cover types were contrasted with the high-resolution satellite images and validated land-use products, and could be adjusted by correcting the samples to improve the classification accuracy.

We conducted the accuracy assessment based on the selected random sample points, which were selected via visual interpretation and validated multi-source land-cover data. The sample-based accuracy evaluation for each obtained Landsat composite was calculated by constructing a confusion matrix, which included the overall accuracy, kappa coefficient, user’s accuracy (UA), and producer’s accuracy (PA). Meanwhile, a comparative analysis of different types of classifiers was performed with the above-mentioned bands as inputs, as shown in Supplementary Table S1.

#### 2.3.4. Multiple Linear Stepwise Regression Analysis

The multiple linear stepwise regression method was adopted to explore the key factors driving the LULC changes, which were determined using a repeated iterative regression analysis. Here, the areas of different land-cover types within the selected periods were used as the dependent variables and the average annual natural and socioeconomic factors were used as independent variables to conduct multiple linear stepwise regression analyses. In this study, the areas of water bodies in relation to the environmental variables (mainly runoff and average precipitation during the dry season) were assessed. Similarly, the response of the three types of land (built-up area, vegetation, and barren) to socioeconomic factors (the total population at the end of the year; gross domestic product (GDP); the proportion of primary, secondary, and tertiary industries; the fixed capital of the whole society; and the urbanization rate from the three aspects of population, economic growth, and urbanization development) was assessed. Furthermore, other factors (human factors and government’s land-use policies) were discussed. 

## 3. Results

### 3.1. Accuracy Assessment

As shown in Table 2, the parameters of the confusion matrix for all six classified composites in their respective periods were calculated; the accuracy assessment yielded an overall accuracy ranging from 88% to 92%. The surface water and vegetation categories showed high accuracies, with the UA over 93% and PA over 94%. Compared with surface water and vegetation, the built-up areas and barren areas showed a relatively low accuracy. It was difficult to distinguish between built-up and barren areas, mainly because of their similar spectral characteristics [21]. Although barren land had a lower PA, the overall accuracy was comparatively high for all classification periods, which indicated a strong consistency between the four land-cover types and their corresponding validation datasets. Overall, the accuracies of the period-specific classification images were sufficient to evaluate the regional land-cover patterns and analyze the land-use change.

### 3.2. Spatial Distribution of Land Cover

**Figure 4:**
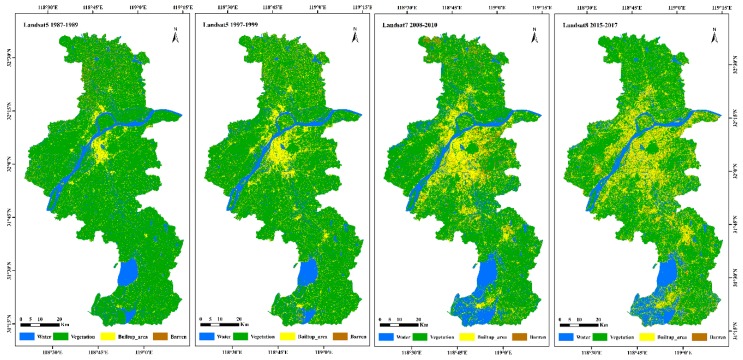
Four selected thematic land-cover maps of the Nanjing region in the lower reaches of the Yangtze River created in the Google Earth Engine (GEE) for 1987–1989, 1997–1999 (using Landsat 5 imagery), 2008–2010 (using Landsat 7 imagery), and 2015–2017 (using Landsat 8 imagery).

Thematic land-cover maps that were representative of six three-year periods spanning 30 years from 1987 to 2017 were generated using the RF classification method for the central area of the lower reaches of the Yangtze River. Four classified composites, one per decade (1987–1989, 1997–1999, 2008–2010, and 2015–2017), were selected and are shown in Figure 4, accompanied by the calculated areas of the different land-cover classes using the algorithm “ee.Image.pixelArea()” in Table 3. Moreover, the total areas occupied by different land-cover types of the six classified composites were graphed to explore the variation trends of different ground object types over the past 30 years, as shown in Supplementary Figure S2.

It can be seen that the urbanization of the Nanjing region presents a multi-core and multi-center structure model radiating to the surrounding area, with the built-up area spreading around different centers. Over the past 30 years, the built-up area has continued to expand, increasing from 537.85 km^2^ to 1500.54 km^2^, especially during the periods between 1987–1989 and 1997–1999, where it increased rapidly at a rate of 71.62%. The total area occupied by the built-up area expanded by 178.9% to occupy an additional 962.69 km^2^. Water decreased first, then increased, and generally showed an increasing trend, expanding by 17.94%, with an increased area of approximately 131 km^2^. Barren land accounted for 6.56% of the total area in the period of 2015–2017, which was an increase of 94.84% throughout 1987–1989. The expansion of the built-up area was accompanied by an overall 25.56% (1305.70 km^2^) reduction in vegetation. The vegetated area continued to decrease at a high rate, with a 13.65% reduction between 1997–1999 and 2008–2010.

### 3.3. Spatiotemporal Changes of Land Cover

**Figure 5:**
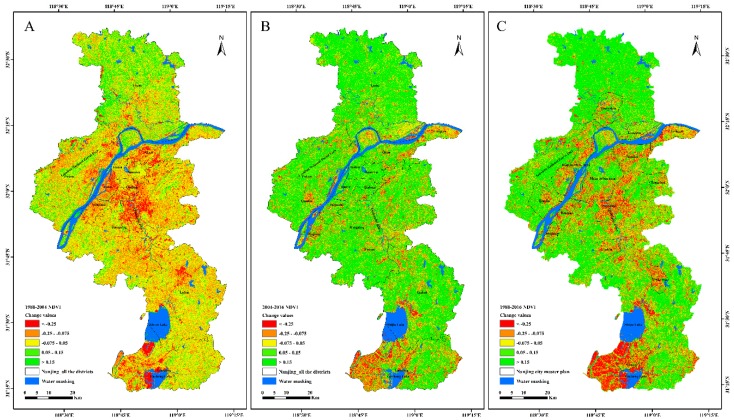
Spatial distribution of the normalized difference vegetable index (NDVI) change in Nanjing with the minimal surface water as the masking in the period 1987–2017: (**A**) NDVI change during the period from 1988 to 2004, (**B**) NDVI change during the period 2004–2017, and (**C**) NDVI change during the period from 1988 to 2016.

To further analyze the changes of NDVI in vegetation over the past 30 years, NDVI change maps of vegetation over two periods were created using an overlay analysis of three composite images, as shown in Figure 5. During the period from 1988 to 2004, the regional greenness decreased significantly, especially around the central area initially composed of Gulou District, Qinhuai District, and Xuanwu District. The reduction of vegetation was specifically distributed in Jianye District, Yuhuatai District, the southwest of Qixia District, the southeast of Laoshan National Forest Park in Pukou District, and parts of areas where Qinhuai River flowed through Jiangning District. In addition, the vegetation index values in southern Shijiu Lake and western Gucheng Lake in Gaochun District also displayed a significant downtrend. Meanwhile, the NDVI change in the northern part of Liuhe District increased, but this was not obvious. During the period 2004–2017, the vegetation index values generally showed an upward trend, even in the central urban areas, which reflected the fact that the government started to pay attention to urban greening while developing the social economy. The areas where the NDVI values declined were mainly distributed along the banks of the Yangtze River Basin; in the construction areas of some new towns, such as Longtan, Qiaolin, and Lukou; and the southwestern part of Gaochun District surrounded by Shijie Lake and Gucheng Lake, as shown in Figure 5B. Figure 5C shows the overall NDVI change over the past 30 years. Regions with a large increase in the vegetation index (>0.15) were mainly located in the north of Liuhe District, the west of Pukou District, the southwest of Jiangning District, the eastern part of Lishui District, the center of Gaochun District, and some low mountain and hilly areas. The areas with a significant decrease in the vegetation index (<−0.25) were mainly distributed in the new urban areas built according to the 2011–2020 Nanjing Urban Master Plan, where vegetation was mainly converted to built-up and barren areas, and the southwest region of Gaochun District with a dense network of rivers, where vegetation was transformed into water bodies.

**Figure 6:**
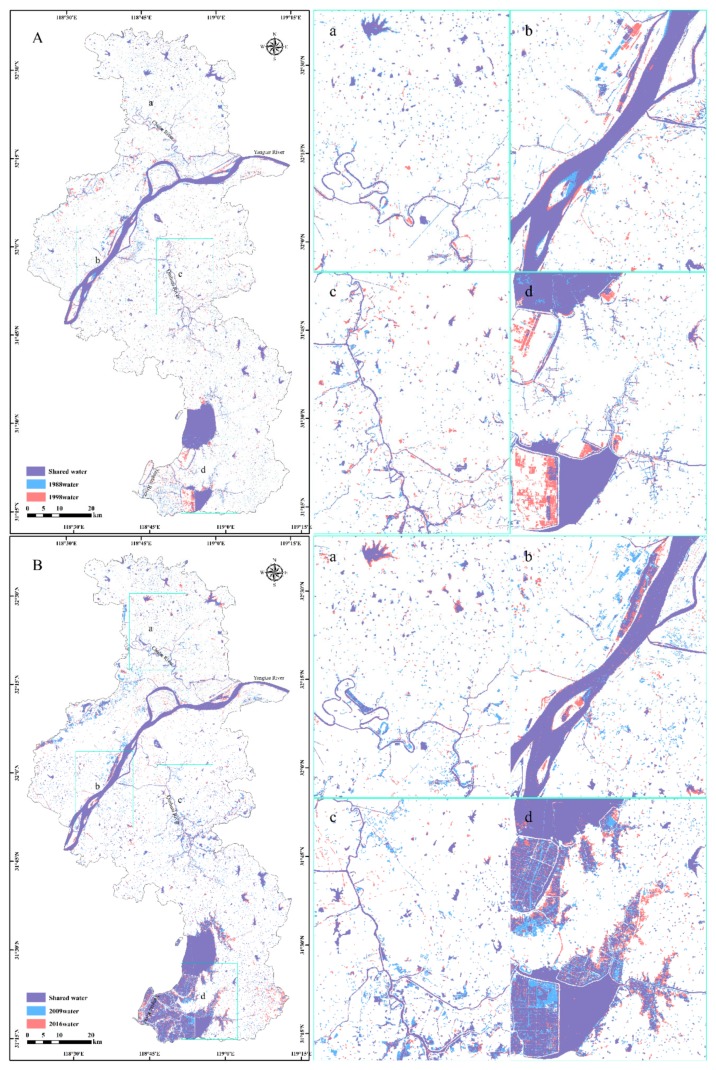
Spatial distribution and variation of surface water in the periods 1987–1989 (**A**), 1997–1999 (**A**), 2008–2010 (**B**), and 2015–2017 (**B**); a, b, c, and d show the enlarged areas in the Chuhe River system (ChRS), the Yangtze River Nanjing section (YRNS), the Qinhuai River system (QhRS), and the Shuiyang River system (SyRS), respectively.

To minimize the influence of seasonal fluctuations in assessing the long-term water changes, in addition to the selection of times for image collections, only relatively large water areas (>40 ha) were considered, following the guidelines provided by Smith et al. [42]. This was done because rivers, streams, and lakes smaller than 40 ha, as well as lakes with direct surface connections to water systems, were more vulnerable to seasonal climate change than larger closed-basin lakes [44]. After eliminating the water bodies smaller than 40 ha, the spatial distributions of surface water for the periods 1987–1989, 1997–1999, 2008–2010, and 2015–2017 were produced and are presented in Figure 6.

**Figure 7:**
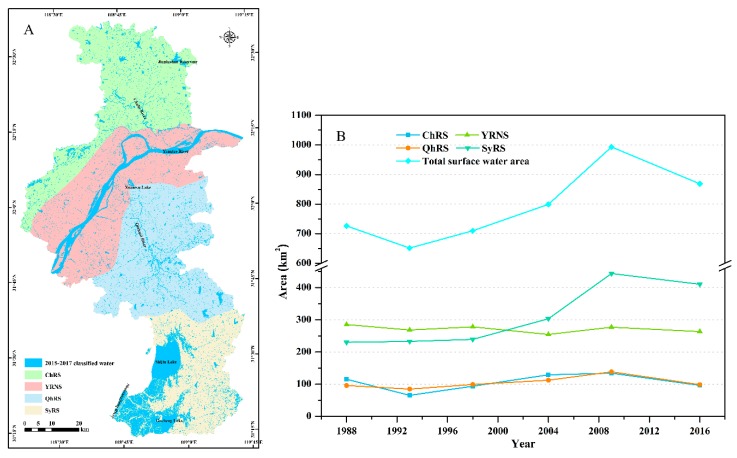
Dynamic changes of surface water extents in four sub-basins: (**A**) division of the water system within the study area and (**B**) areal changes in surface water extent in all four sub-basins during the period 1987–2017.

The Yangtze River system covered most of the study area (accounting for 95.49%) and was subdivided into four water systems. From north to south, these were the Chuhe River system (ChRS), the Yangtze River Nanjing section (YRNS), the Qinhuai River system (QhRS), and the Shuiyang River system (SyRS), as shown in Figure 7A. Lakes and reservoirs were scattered with numerous rivers in Nanjing. To further quantify the hydrologic changes, the areal extent of surface water in the four sub-basins for each of the periods were calculated, as shown in Figure 7B.

To analyze the change and transformation of LULC types, the land-use transition matrix between 1987–1989 and 1997–1999 was derived using the overlay function in ArcGIS 10.2 (Esri, BeiJing, China), as well as the land-use transfer matrix between 1997–1999 and 2015–2017, as shown in Supplementary Table S2. Supplementary Figure S3 presents the transformation of land-use categories during the period from 1987 to 2017. During the period 1987–1999, 87.1% of vegetation (4446.3 km^2^) remained unchanged, 8.0% of vegetation (407.9 km^2^) was converted into built-up areas, and 3% of vegetation (155.3 km^2^) was converted into barren land; during the period 1999–2017, 14.7% of vegetation (687.62 km^2^) was converted into built-up areas, 6.6% of vegetation (307.0 km^2^) was converted into barren land, and 5.8% of vegetation (272.1 km^2^) was transformed into water bodies. For water bodies, during the period 1987–1999, 78.0% of water (567.8 km^2^) remained unchanged, 5.9% of water (43.2 km^2^) was transformed into built-up areas and 11.5% of water (83.7 km^2^) was converted into vegetation; during the period 1999–2017, 9.4% of water (67.1 km^2^) was converted into built-up areas, 13.0% of water (92.4 km^2^) was converted into vegetation, and 3.7% of water was transformed into barren land. For the built-up area, during the period 1987–1999, 76.7% of the built-up area (412.6 km^2^) remained unchanged, 10.3% of the built-up area (55.4 km^2^) was transformed into vegetation, and 7.9% of the built-up area (49.71 km^2^) was converted into barren land; during the period 1999–2017, 71.3% of the built-up area (656.9 km^2^) remained unchanged, 8.0% of the built-up area (73.6 km^2^) was transformed into barren land, and 18.8% of the built-up area (173 km^2^) was transformed into vegetation. For barren land, during the period 1987–1999, 32.1% of barren land (59.4 km^2^) was converted into built-up areas and 44.5% of barren land (98.9 km^2^) was transformed into vegetation; during the period 1999–2017, 32.1% of barren land (89.0 km^2^) was converted into built-up areas and 43.2% of barren land (119.7 km^2^) was converted into vegetation. In general, during the period 1987–1999, the significant increase of the built-up area (385.2 km^2^) was mainly due to a transformation from vegetation (352.5 km^2^), and the apparent reduction of vegetation (423.7 km^2^) was mainly due to its conversion to built-up areas (368.3 km^2^) and barren land (56.5 km^2^). During the period 1999–2017, the increase of built-up area (579.2 km^2^) was mainly due to a transformation from vegetation (514.6 km^2^), while the reduction of vegetation (881.6 km^2^) was mainly due to its conversion to built-up areas (514.6 km^2^), barren land (187.3 km^2^), and water bodies (179.8 km^2^).

**Figure 8:**
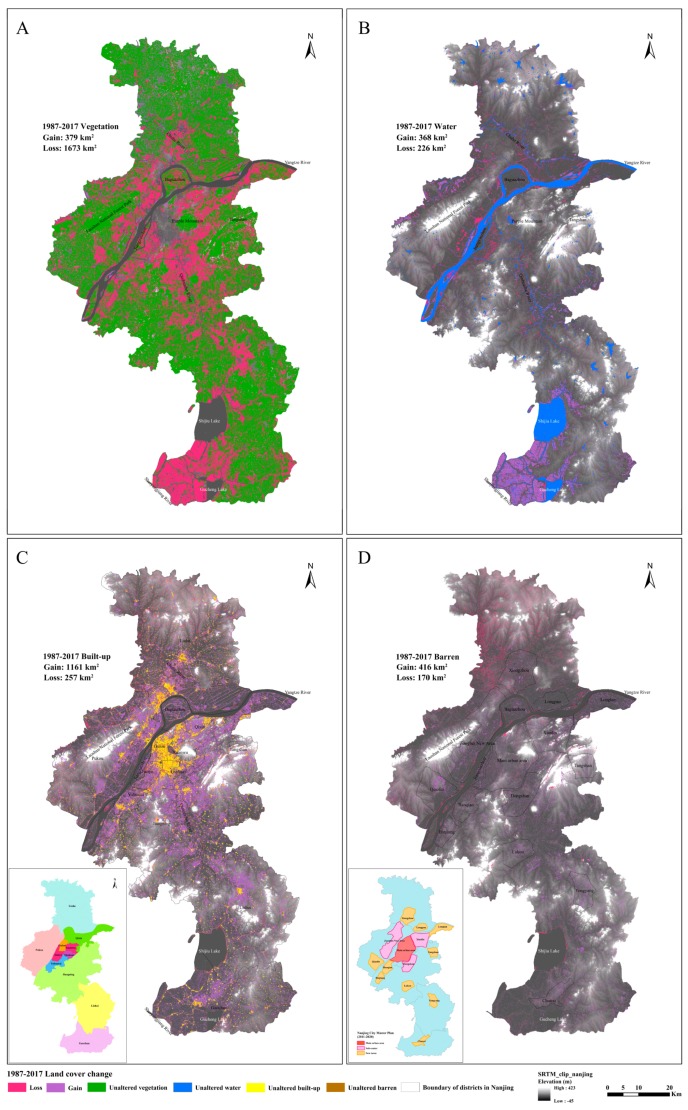
Spatial distribution of land-use/land-cover (LULC) change between the classified composites in 1987–1989 and 2015–2017 using a grayscale topography (SRTM 30 m spatial resolution): (**A**) vegetation, (**B**) water, (**C**) built-up area (the illustration is the administrative division of Nanjing), and (**D**) barren land. The lower-left corner shows the master plan of Nanjing. Loss indicates that the land-cover category was converted to other land-cover categories, while Gain shows that other land-use types were transferred to the given land-cover type.

To explore the spatial pattern of LULC changes between 1987–1989 and 2015–2017, maps depicting changes in land-cover categories were compiled. For this purpose, the composites of these two periods were converted into vector data and then the joint tool in ArcGIS was used to obtain the changes of different land-use types, as shown in Figure 8. The administrative divisions of each district in Nanjing are shown in the illustration in Figure 8C. Vegetation showed a significant reduction (decrease of 1285.7 km^2^, 25.2% of the initial vegetation area; Figure 8A). The area of vegetation loss was 1552.67 km^2^ and was mainly distributed in the areas surrounding the main urban area and southwest of Nanjing, including the north and south banks of the Nanjing section of the Yangtze River, the Qinhuai River Basin, the Chuhe River Basin, and southwest of Gaochun District. In addition, the vegetation of Jiangxinzhou and Baguazhou in the center of the Yangtze River also decreased by varying degrees. The area of vegetation gain was 378.7 km^2^ and was mainly distributed in the north of Liuhe District and the west of Pukou District.

Water bodies generally showed an increasing trend (increase of 130.6 km^2^, +18%; Figure 8B). The area of water loss was 225.7 km^2^ and was mainly distributed along the banks of the Yangtze River and Chuhe River, especially in the southeast of Laoshan National Forest Park and in Jianye District of Nanjing. The formation of central bars in the Nanjing section of the Yangtze River also indirectly led to the reduction of the water body area, as well as the shrinkage of lakes and wetlands throughout the study area. The area of water gain was 367.9 km^2^ and was mainly distributed in the southwest of the study area, especially the western part of Gaochun District, which was surrounded by Gucheng and Shijiu Lakes with dense river networks belonging to the Shuiyang River system.

The built-up area showed a rapid and large-scale expansion (962.7 km^2^, +178%; Figure 8C). The original main urban area was based on the Yangtze River, mainly including the Gulou District, Xuanwu District, and Qinhuai District. The peripheral towns developed rapidly as an extension of the development of the main urban area. The area of built-up gain was 1161 km^2^ and was mainly distributed along the north bank of the Yangtze River in Pukou District, along the Qinhuai and Lishui River in Jiangning District, along the Chuhe River in the south of Liuhe District, and in the northern part of Gucheng Lake in Gaochun District. The cause for the loss of built-up area mainly came from the demolition of urban and rural houses according to the urban and rural planning, which aimed at the rational allocation of land and space resources, including urban reconstruction, township transformation, and the new countryside construction. The area of built-up loss reached 257.2 km^2^ and was mainly distributed in the main urban area and Liuhe District.

The barren area increased rapidly compared with that in 1987–1989 (246.1 km^2^, +132%) and the spatial distribution was relatively dispersed (Figure 8D). The area of barren loss was 170 km^2^ and was mainly distributed in the northwest of Liuhe District and southwest of Pukou District, which was attributed to exploitation and utilization, combined with ecological development and greening construction. The area of barren gain was 416.1 km^2^ and was mainly distributed in areas other than the main urban area, where the sub-city areas under construction were located, according to the Nanjing City Master Plan (2011–2020), such as Jiangbei New Area, Dongshan, Xianlin, Lishui, and Gaochun sub-city areas.

## 4. Discussion

### 4.1. Land-Cover Changes Influenced by Natural and Human Factors

An analysis of the LULC change in Nanjing city in the central region of the lower Yangtze River indicated a significant change in the built-up area over the last 30 years. The most notable land-use transformation detected in the study area was the reduction of vegetation and the rapid conversion to built-up areas. To explore the main factors driving the changes in different land-use/land-cover types, multiple linear stepwise regression analysis was applied to identify the mechanisms of land-cover changes.

For water bodies, the regional surface water extent (permanent surface water) mainly reflected the surface water conditions of rivers, lakes, and reservoirs during the dry season, from November to March of the next year, which was less affected by the variations in annual precipitation. Moreover, as the main water system in the study area, the Yangtze River covered all districts and counties, and as the area of the basin accounted for 95.49% of the total land area in Nanjing, it was speculated that runoff in the dry season might be related to the regional surface water extent. Given this, multivariate linear regression of regional surface water, runoff, and precipitation in the dry season was carried out, and the results showed that the key factor driving the changes in regional surface water extent was changes in the runoff of the Yangtze River (R^2^ = 0.87 and *p*-value = 0.054) during the dry seasons from 1987 to 2010. Some other external human factors in different periods also had an impact on surface water changes. Since the first impoundment of the Three Gorges Project in 2003, the regulating role of the Three Gorges Project in the water supply of the Yangtze River and its gradual enhancement of water storage capacity had led to an increase in runoff during the dry season, and the Three Gorges Reservoir experienced the largest flood after its completion in 2010.

Additionally, the project of diverting water from the Yangtze River to the Hanjiang River was undertaken to alleviate the water decrease in the middle and lower reaches of the Hanjiang River caused by the water diversion from the middle route of the South-to-North Water Diversion Project to the cities in northern China in 2014. The East Route of the South-to-North Water Transfer Project began to divert water in December 2013. These two events might be the reason for the decrease of regional surface water in Nanjing within the period of 2015–2017, as shown in Supplementary Figure S2B.

Due to urbanization, the distribution of surface water in the Gulou and Jianye Districts decreased significantly from 1987 to 1999, as well as the distribution of surface water in Pukou District, as shown in Figure 6A,B, respectively. The reduction of surface water along the rivers was largely related to human activities, such as landfilling; converting water and wetlands into built-up areas; and using it as industrial, mining, and construction land, as shown in Figure 6(Ba–Bc), respectively. Nanjing had been vigorously developing aquaculture in recent years, especially since China joined the World Trade Organization (WTO) in 2001, and hence more and more farmland has been converted into fish ponds to meet the increasing demand for aquaculture. The most obvious changes in these water bodies were distributed in the south of Shijiu Lake and west of Gucheng Lake in Gaochun District, as shown in Figure 6(Ad,Bd). Wang et al. [45] adopted a water change intensity index and the regional center of gravity to explore the spatial trajectory of the water area in Nanjing city. They found that the center of gravity of the spatial distribution of water had clearly moved southward since 2006, which could also be explained by the conversion of cultivated land to water bodies in the Lishui and Gaochun Districts. In addition, the spatial and temporal variation characteristics of water bodies in Nanjing were consistent with our research results. However, the dynamic change of water were only discussed in terms of the impacts of local precipitation, urban land expansion, and aquaculture development, but ignored that the study area belonged to the Yangtze River Basin, and did not pay attention to the role of runoff on water dynamic change.

The socio-economic factors affecting the change in the built-up area are complicated; therefore, selecting the appropriate driving factors is the key to analyzing the change of the built-up area. Zhou et al. [46] analyzed the variation of the land-use structure in Nanjing from 1985 to 2002 and only selected the population, GDP, and share of primary industry to explore the driving factors of cultivated land and the built-up area. Here, we selected the total population at the end of the year; population density; GDP; the proportion of primary, secondary and tertiary industries; the fixed capital of the whole society; and the urbanization rate in terms of the three aspects of population, economic growth, and urbanization development. Multiple linear regression analysis results showed that the main factors driving changes in built-up areas were the total population and the proportion of secondary and tertiary industries contributing to GDP:

*Y* = 27.56*X*_1_ + 337.36*X*_2_ − 3508.6, (1)

where *Y* refers to the built-up area (km^2^), *X*_1_ represents the proportion of secondary and tertiary industries, and *X*_2_ represents the total population (million people). As the population increased rapidly, cities and towns expanded to suburbs, and large amounts of agricultural land and other land-cover types were converted into construction land. Meanwhile, industrialization led to an adjustment of the three industrial structures. The decline of the economic status of the first industry made it more difficult to protect the cultivated land, which led to the occupation of cultivated land. Furthermore, the demand for land resources increased rapidly with the development of secondary and tertiary industries. In addition, with the improvement of urban traffic and the guidance of government policies, the original industries in the central area of the city, especially those with labor-intensive and heavy pollution, moved to the suburbs. The rapid rise of industrial parks in the suburbs also stimulated the growth of built-up areas.

The key factors driving the changes in vegetation were GDP, the total population, and the total investment in fixed assets. The expression for the relationship was as follows:

*Y*_1_ = −639.2*X*_2_ − 1.9*X*_3_ + 0.2*X*_4_ + 8161.6, (2)

where *Y*_1_ refers to the vegetation area (km^2^), *X*_2_ represents the total population (million people), *X*_3_ represents the total investment in fixed assets (billion dollars), and *X*_4_ represents GDP (billion dollars). As mentioned above, the increase in population greatly stimulated the expansion of urban and rural construction land. Furthermore, the intensity of engineering construction had increased due to the social and economic development since 2004, such as industrial and mining, energy, and transportation construction, among others, which was also driven by the investment in fixed assets. The amount of vegetation was negatively correlated with GDP, which could be explained by the fact that the output value of agriculture and forestry was far less than that of the secondary and tertiary industries, such as industrial production and real estate development. Therefore, the transformation of vegetation land to these industries promoted the growth of GDP to some extent. The dynamic changes of the main factors driving the changes in the built-up area and vegetation are shown in Supplementary Figure S4. All these factors led to a dramatic reduction in vegetation and its transformation into built-up areas, which was consistent with the change in the land-cover transfer matrix. In recent years, in accordance with the development requirements of modern agriculture, Nanjing has accelerated the adjustment of the agricultural industrial structure and the scale of fishery output has increased year by year. Fishery’s share of total agricultural production increased from 6.5% in 1990 to 21.3% in 2009, and the most obvious response to land use was the conversion of vegetation to water bodies around Shijiu Lake and Gucheng Lake, as shown in Figure 8A,B, respectively.

The contributions of the above-mentioned factors that drove the changes in barren land were not obvious (Supplementary Table S3). However, barren land was related to the development environments and land policies within the study area, which could be inferred from the dynamic change and distribution of the barren land mentioned above. Nanjing vigorously developed a township industry, where a large amount of cultivated land was occupied in the late 1980s, accompanied by the increase of barren land. The urban planning law enacted in 1989 clearly proposed that urban system planning should be formulated. However, due to the overheated economy in the early 1990s, urbanization went through a period of rapid development with the deepening of economic reform, development zones were established everywhere to develop real estate, and the phenomenon of losing control of cultivated land appeared again. The uncultivated land and barren land in Jiangbei District (especially in Liuhe District) were developed and mainly transformed into built-up areas in accordance with the requirements of the comprehensive and integrated development of the Nanjing city overall planning adjustment (1991–2010). Furthermore, barren land was accompanied by the construction of sub-cities and new towns in accordance with the Nanjing City Master Plan (2011–2020), as shown in Figure 8D.

### 4.2. Advantages and Limitations of Using the GEE

The accuracy evaluation of the research results showed that the proposed methods for image mosaicking and sample selection can be quickly and accurately used for land-cover mapping via the GEE platform. The advantages of using the GEE in this work are mainly reflected in the following three aspects: First, the GEE is a free and web-based cloud-computing platform, which offers more robust processing power and saves computing time more effectively, compared with traditional image processing tools. Moreover, it is constructed based on Google Cloud and can be shared via URLs and thus is not limited by time and place. Second, the GEE provides accessibility to 11 PB of earth observation data and many powerful servers, such as the Landsat archives, DMSP-OLS, and DEM products, along with other types of satellite datasets, without the need to retrieve or download large volumes of spatiotemporal data, which greatly reduces the time required for preparations. Third, the script stored in the GEE can be easily optimized and applied for the long-term monitoring of the LULC changes.

However, there are a few limitations regarding using the GEE in image preprocessing and sample selection. The algorithm is not perfect and needs to be improved. For the damaged stripe in the Landsat 7 ETM data products after 31 May 2003, the GapFill function utilized on the GEE platform did not work as well as in local remote sensing software, as there was still a relatively distinct streak even though the missing stripe was repaired. When selecting samples directly on the GEE platform, it was difficult to control the positional accuracy of the sample points, which needed to be further processed in ArcGIS. Therefore, extra effort was required to download the preprocessed composite images for sample selection and upload the selected sample vector data for image classification. Furthermore, algorithms created in the Google Earth Engine run in the Google cloud, where debugging can be challenging because errors can occur either in the client-side JavaScript code or the server-side execution of the coded instructions. For example, when calculating the pixel area in the GEE in this article, the land-cover categories were first binarized, then multiplied by the ee.Image.PixelArea(), and finally, we summed the result of the multiplication with the ee.Reducer.sum(). Among them, the scale parameter should be set to 30 for the Landsat images, but it occasionally took a significant amount of time to finish the computation such that a “computation time out” error might be thrown. If the scale parameter was increased to allow for the computation to succeed, there would be a deviation in the area calculation results. Hence, it was better to use GIS for geospatial data analysis when the area could not be calculated with a scale parameter of 30. In addition, the GEE lacks follow-up image analysis algorithms to monitor the land-cover change, which means that the classification results needed to be downloaded when further analysis was required.

## 5. Conclusions

This study focused on the status and trends of land-cover change in the central region of the lower Yangtze River over the past 30 years. Based on the constructed sample datasets with sufficient auxiliary data, a multi-data composite technique was performed by utilizing dense stacks of all available Landsat imagery to generate inter-annual updated land-cover products, combined with the using of an expanded RF algorithm. This proposed methodology for monitoring long-term urban land-cover changes using the GEE, when combined with an in-depth understanding of the influence of natural and socioeconomic factors on urban land-cover changes, can be used for urban land administration and planning. Additionally, the results obtained with this method can help us understand the context of urbanization and economic growth. Studying and mastering the dynamic changes of urban LULC in Nanjing will not only help to understand the natural environmental factors in the city’s ecosystem and the comprehensive impact of human activities on land-use changes, but also provide references for the regional use of surface land resources in similar areas.

## Figures and Tables

**Table 1:** The selected image collections for the study area.

Satellite Sensor	Three-Year Period	Date Frame	Image Count
Landsat 5	1987–1989	1 Mar to 15 Jun and 16 Jul to 31 Nov	36
Landsat 5	1992–1994	1 Mar to 15 Jun and 16 Jul to 31 Nov	39
Landsat 5	1997–1999	1 Mar to 15 Jun and 16 Jul to 31 Nov	26
Landsat 7	2003–2005	1 Mar to 15 Jun and 16 Jul to 31 Nov	40
Landsat 7	2008–2010	1 Mar to 15 Jun and 16 Jul to 31 Nov	52
Landsat 8	2015–2017	1 Mar to 15 Jun and 16 Jul to 31 Nov	30

**Table 2:** Accuracy assessment of the land-cover classification results in the periods 1987–1989, 1992–1994, 1997–1999, 2003–2005, 2008–2010, and 2015–2017.

Three-Year Period	Accuracy	Surface Water	Vegetation	Built-Up Area	Barren
1987–1989	User’s	0.95 ± 0.02	**0.99 ± 0.01**	0.85 ± 0.02	*0.84 ± 0.04*
	Producer’s	0.95 ± 0.01	**1 ± 0.00**	0.92 ± 0.04	*0.65 ± 0.04*
	Overall	0.90 ± 0.01			
	Kappa	0.86 ± 0.01			
1992–1994	User’s	0.95 ± 0.02	**0.98 ± 0.01**	0.84 ± 0.02	*0.85 ± 0.01*
	Producer’s	0.95 ± 0.01	**1 ± 0.00**	0.90 ± 0.01	*0.75 ± 0.03*
	Overall	0.89 ± 0.01			
	Kappa	0.85 ± 0.02			
1997–1999	User’s	0.95 ± 0.02	**0.99 ± 0.00**	0.85 ± 0.00	*0.86 ± 0.01*
	Producer’s	0.96 ± 0.02	**1 ± 0.00**	0.91 ± 0.01	*0.70 ± 0.06*
	Overall	0.90 ± 0.02			
	Kappa	0.87 ± 0.01			
2003–2005	User’s	0.99 ± 0.01	**0.99 ± 0.01**	0.82 ± 0.03	*0.83 ± 0.04*
	Producer’s	0.98 ± 0.01	**0.99 ± 0.01**	0.84 ± 0.01	*0.79 ± 0.04*
	Overall	0.89 ± 0.01			
	Kappa	0.85 ± 0.02			
2008–2010	User’s	**0.99 ± 0.01**	0.97 ± 0.02	0.84 ± 0.02	*0.83 ± 0.01*
	Producer’s	**0.98 ± 0.02**	0.97 ± 0.01	0.84 ± 0.04	*0.82 ± 0.04*
	Overall	0.90 ± 0.005			
	Kappa	0.87 ± 0.005			
2015–2017	User’s	**0.99 ± 0.01**	0.98 ± 0.01	0.78 ± 0.05	*0.84 ± 0.03*
	Producer’s	**1 ± 0.00**	0.97 ± 0.00	0.84 ± 0.04	*0.76 ± 0.06*
	Overall	0.90 ± 0.02			
	Kappa	0.86 ± 0.02			

Note: The best classification results are indicated in bold font, and the worst classification results are indicated in italic font.

**Table 3:** Land-cover and land-use change from 1987 to 2017.

Period	Land Area	Water	Vegetation	Built-Up Area	Barren
1987–1989	Area (km^2^)	728.01	5107.89	537.85	222.08
	Percentage	11.04	77.44	8.15	3.37
	Change rate (%)	-	-	-	-
1997–1999	Area (km^2^)	711.48	4684.17	923.05	277.12
	Percentage	10.79	71.02	13.99	4.20
	Change rate (%)	−2.27	−8.30	71.62	24.78
2008–2010	Area (km^2^)	993.10	4044.74	1212.81	334.42
	Percentage	15.06	61.32	18.39	5.07
	Change rate (%)	39.58	−13.65	31.39	20.68
2015–2017	Area (km^2^)	858.58	3802.19	1500.54	432.72
	Percentage	13.02	57.66	22.76	6.56
	Change rate (%)	−13.55	−6.00	23.72	29.39

## Supplementary Materials

The following are available online at https://www.mdpi.com/1424-8220/20/7/2091/s1, Figure S1: Impacts of different numbers of trees in the random forest (RF) based on the overall accuracy and kappa coefficient, Figure S2: Variation trends of different ground object types over the past 30 years: (A) vegetation, (B) water, (C) built-up area, (D) barren land, Figure S3: Transformation of the land-use/land-cover (LULC) types during the period 1987–2017: (A) land-use change between 1987–1989 and 1997–1999, and (B) land-use change between 1997–1999 and 2015–2017, Figure S4: The dynamic changes of the main factors driving the changes in the built-up area and vegetation during the period 1987–2017: (A)Total population, (B) GDP, (C) The proportion of secondary and tertiary industries’ contribution to the GDP, (D) Total investment in fixed assets. Table S1: Comparative analysis and evaluation of different classification algorithms for the Landsat 8 composite image during the period 2015–2017, Table S2: Land use transfer matrices between 1987–1989, 1997–1999, and 2015–2017, Table S3: Correlation coefficient of land use change and its driving factors in Nanjing.
