# Supplementary material for: Land Cover Change in the Central Region of the Lower Yangtze River Based on Landsat Imagery and the Google Earth Engine: A Case Study in Nanjing, China
Source: Sensors (Basel). 2020 Apr 8;20(7):2091. doi: 10.3390/s20072091 (PMC7180629; doi:10.3390/s20072091)
Supplement: Supplementary file 1 [file sensors-20-02091-s001.pdf]

Appendix A. Supplementary Material

Land Cover Change in the Central Region of the Lower Yangtze River Based on Landsat Imagery and the Google Earth Engine: A Case Study in Nanjing, China

Dong-Dong Zhang [1] and Lei Zhang [2,*]

1 Shanghai Key Laboratory of Multidimensional Information Processing, East China Normal University, Shanghai 201100, China; 52161213021@stu.ecnu.edu.cn
2 MOE International Joint Lab of Trustworthy Software, East China Normal University, Shanghai 200062, China
* Correspondence: lzhang@ce.ecnu.edu.cn; Tel.: +86-188-1822-3050

Contents

1. Scripts for image pre-processing using dense stacks of Landsat imagery, including date filtering, cloud mask and mosaicking, random forest (RF) classification, accuracy assessment, and regional area calculations.
2. The entirety of Landsat 5, 7, and 8 image collections was analyzed using the Tier 1 top of atmosphere (TOA) reflectance product for the study area with the written application of a multi-scene image display spanning from 1987 to 2017.
3. Video for display (comparative analysis regarding the use of dense stacks of Landsat imagery instead of using annual imagery displayed via the normalized difference vegetable index (NDVI) dynamic change).
4. Download link for the Landsat image composites for different periods.
5. Download link for the training sample dataset.
6. Download link for the land-use/land-change (LULC) RF classification results.
7. Scripts for the comparative analysis of different classifiers.

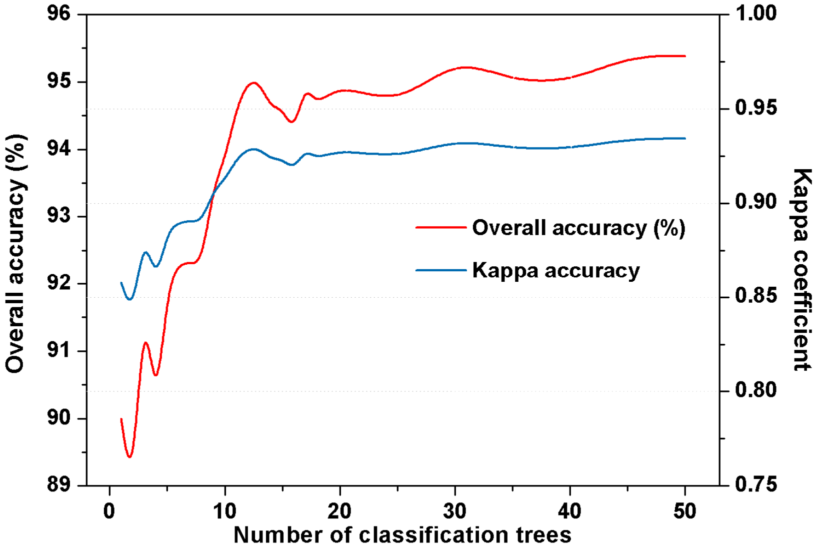

Figure S1. Impacts of different numbers of trees in the random forest (RF) based on the overall accuracy and kappa coefficient.

1. Scripts for image pre-processing using dense stacks of Landsat imagery, including date filtering, cloud mask and mosaicking, RF classification, accuracy assessment, and regional area calculations.
   Links to the Google Earth Engine: l5_1988_classification.
   https://code.earthengine.google.com/a821b9bd81bba7d4691977731ffa722c
   Links to the Google Earth Engine: l5_1993_classification.
   https://code.earthengine.google.com/e6e578b827ba939b1e88d52b387fc0ad
   Links to the Google Earth Engine: l5_1998_classification.
   https://code.earthengine.google.com/8f9ee6cbba4310ad0a553a01b116c728
   Links to the Google Earth Engine: l7_2004_classification.
   https://code.earthengine.google.com/fd5d9f6f96ec4df06b48d3a16006a3c7
   Links to the Google Earth Engine: l7_2009_classification.
   https://code.earthengine.google.com/d2bc795b1103698bd1f22bc7c29bd851
   Links to the Google Earth Engine: l7_2016_classification.
   https://code.earthengine.google.com/cb7f2c3527c9754c7df06353ba42fd12
2. The entirety of Landsat 5, 7, and 8 image collections was analyzed using the Tier 1 top of atmosphere (TOA) reflectance product for the study area with the written application of a multi-scene image display spanning from 1987 to 2017.
   Links to the Google Earth Engine: l5_Multi-scene image display.
   https://code.earthengine.google.com/41d179c222059dc68520a0a57d5c776a
   Links to the Google Earth Engine: l7_Multi-scene image display.
   https://code.earthengine.google.com/6e5a7cd6ce732a544905b6821eee98b9
   Links to the Google Earth Engine: l8_Multi-scene image display.
   https://code.earthengine.google.com/2c04007403c7b6870458f71906bec466
3. Video for display (comparative analysis regarding the use of dense stacks of Landsat imagery instead of using annual imagery displayed via the NDVI dynamic change).
   Links to the Google Earth Engine: video (annual NDVI dynamic change).
   https://code.earthengine.google.com/ae8551739790d7734e7f659c34fe92d9
   Links to the Google Earth Engine: video (dense stacks).
   https://code.earthengine.google.com/d2f50a31683088dafb85c51d24050bde
4. Download link for the Landsat image composites for different periods.
   Links to Google Drive: Landsat image composites within different periods.
   https://drive.google.com/open?id=1vWFYYPF12GRZpcAyp0mN_RIR9g8A0CrF
5. Download link for the training sample dataset.
   Links to Google Drive: Training sample dataset.
   https://drive.google.com/open?id=123cgpJgmXbbxPrWZf3vZjQM78JEF7yl_
6. Download link for the LULC RF classification results.
   Links to Google Drive: LULC RF classification results.
   https://drive.google.com/open?id=1bAYyOIV7rp2nueDNILICmVjCf_AHSzwK
7. Scripts for the comparative analysis of different classifiers.
   Links to the Google Earth Engine: Compare different types of classifiers.
   https://code.earthengine.google.com/9d633e68da1be4a82eddf8d7b460eb97

Table S1. Comparative analysis and evaluation of different classification algorithms for the Landsat 8 composite image during the period 2015–2017.

Classification Methods	Accuracy	Vegetation	Surface Water	Built-Up Area	Barren Land
Cart	User's	0.98	0.96	0.80	0.78
	Producer's	0.96	0.96	0.81	0.87
	Overall		0.88		
	Kappa		0.84		
RF	User's	0.97	0.97	0.87	0.92
	Producer's	0.97	0.93	0.94	0.88
	Overall		0.93		
	Kappa		0.91		
NaiveBayes	User's	0.99	0.96	0.59	0.64
	Producer's	0.96	0.87	0.73	0.56
	Overall		0.79		
	Kappa		0.70		
GmoMaxEnt	User's	0.97	0.96	0.65	0.76
	Producer's	1	0.92	0.81	0.56
	Overall		0.82		
	Kappa		0.76		
Support Vector Machine (SVM)	User's	0.97	0.95	0.87	0.82
	Producer's	0.99	0.93	0.84	0.85
	Overall		0.90		
	Kappa		0.87		

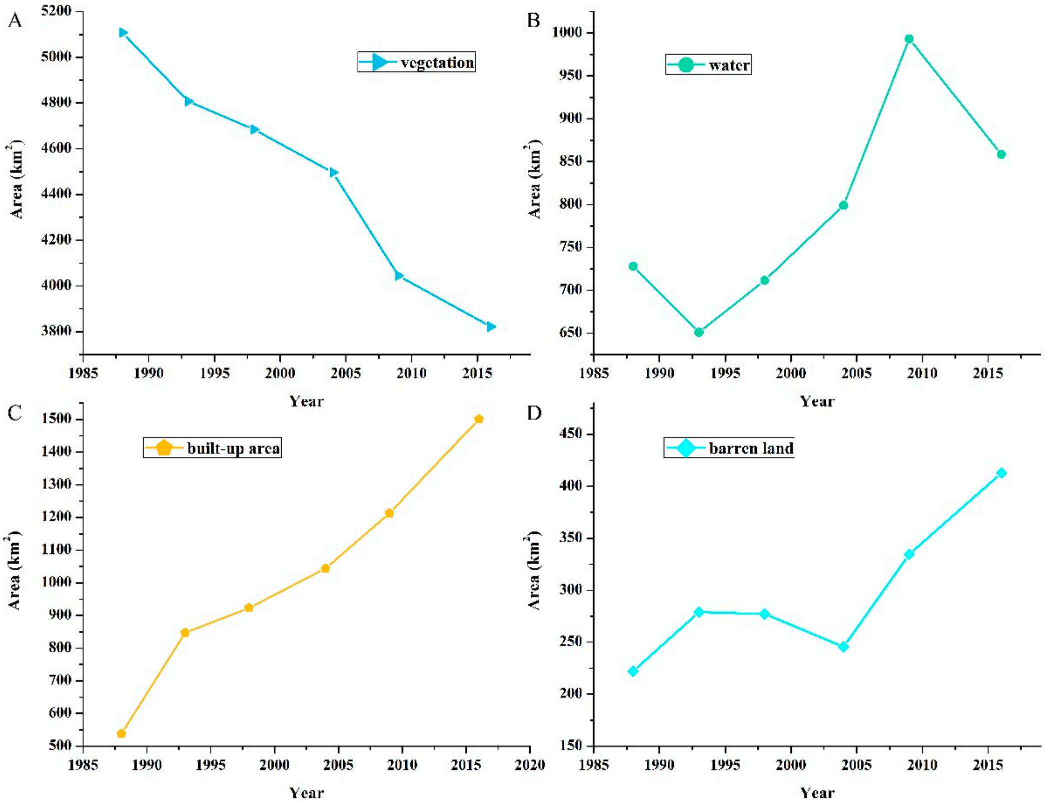

Figure S2. Variation trends of different ground object types over the past 30 years: (A)vegetation,

(B)water, (C)built-up area, (D) barren land.

Table S2. Land use transfer matrices between 1987–1989, 1997–1999, and 2015–2017.

Land-Cover Type	1998_Barren	1998_Builtup_Area	1998_Vegetation	1998_Water	Area
**1988_Barren**	45.85	59.40	98.87	17.96	222.08
**1988_Builtup_Area**	42.53	412.60	55.39	27.32	537.85
**1988_Vegetation**	155.33	407.86	4446.26	98.44	5107.89
**1988_Water**	33.41	43.19	83.65	567.76	728.01
**Area**	277.12	923.05	4684.17	711.48	6595.82

Land-Cover Type	2016_Barren	2016_Builtup_Area	2016_Vegetation	2016_Water	Area
**1998_Barren**	25.78	88.95	119.73	42.67	277.12
**1998_Builtup_Area**	73.64	656.91	173.00	17.80	921.35
**1998_Vegetation**	306.98	687.62	3407.08	272.13	4683.81
**1998_Water**	26.32	67.07	92.38	525.98	711.75
**Area**	432.72	1500.54	3802.19	858.58	6594.03

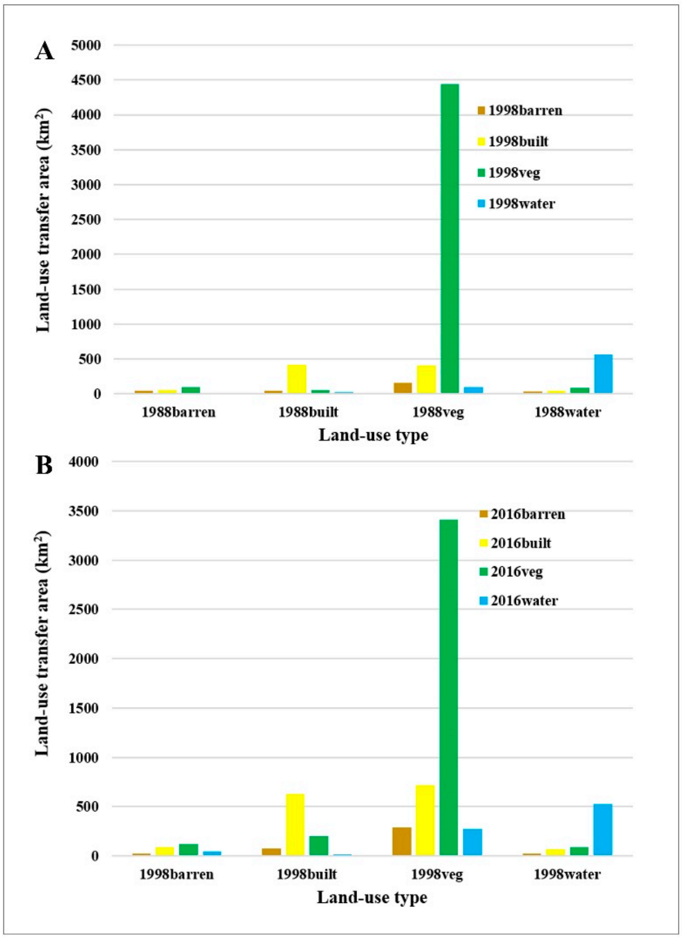

Figure S3. Transformation of the land-use/land-cover (LULC) types during the period 1987–2017: (**A**) land-use change between 1987–1989 and 1997–1999, and (**B**) land-use change between 1997–1999 and 2015–2017.

Table S3. Correlation coefficient of land use change and its driving factors in Nanjing.

Land-Cover Types / Driving Factors	Built-Up Area	Vegetation	Barren Land
Total population	0.975**	−0.982**	-
GDP	0.834*	−0.901*	-
Primary proportion of GDP	−0.963**	0.817*	-
Secondary proportion of GDP	−0.949**	0.918**	-
Tertiary proportion of GDP	0.978**	−0.908*	-
STP	0.963**	−0.820*	-
Total investment in fixed assets	0.856*	−0.933**	-
Urbanization rate	0.951**	−0.953**	-

Note 1: * represents a significant correlation at the level of 0.05, and ** represents a significant correlation at the level of 0.01. Note 2: STP represents the proportion of secondary and tertiary industries' contribution to the GDP.

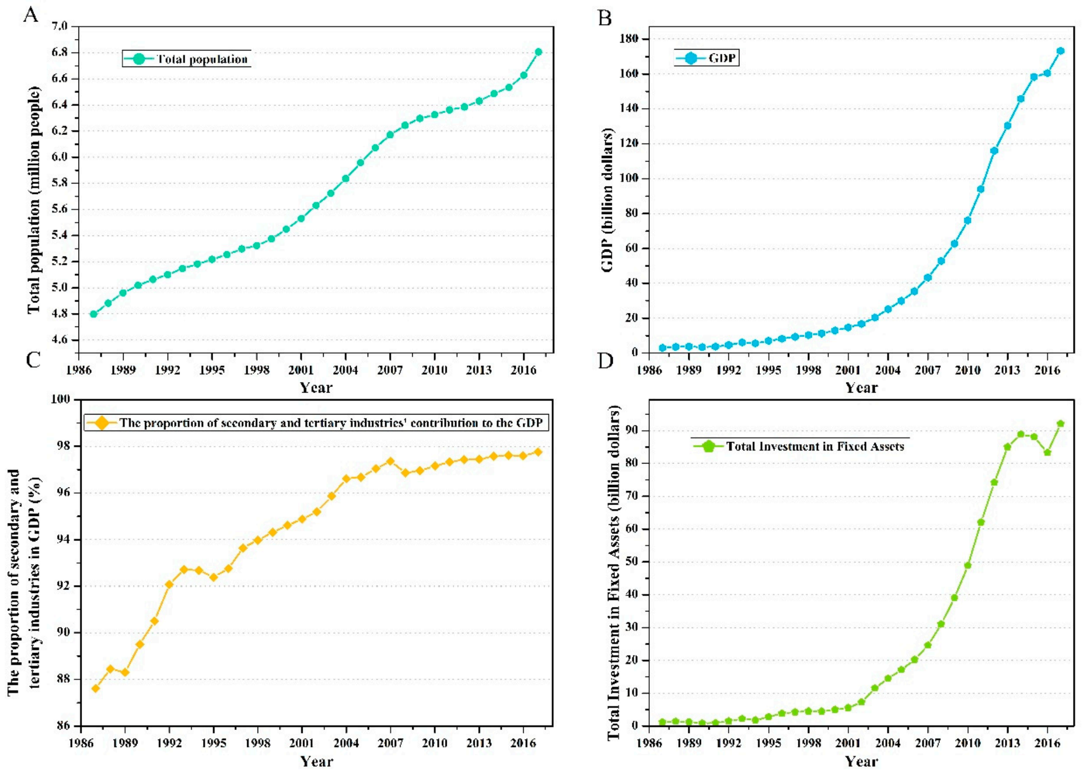

Figure S4. The dynamic changes of the main factors driving the changes in the built-up area and vegetation during the period 1987–2017: (A)Total population, (B) GDP, (C) The proportion of secondary and tertiary industries' contribution to the GDP, (D) Total investment in fixed assets.
